# Circulating Endocannabinoids and the Polymorphism 385C>A in Fatty Acid Amide Hydrolase (*FAAH*) Gene May Identify the Obesity Phenotype Related to Cardiometabolic Risk: A Study Conducted in a Brazilian Population of Complex Interethnic Admixture

**DOI:** 10.1371/journal.pone.0142728

**Published:** 2015-11-11

**Authors:** Cyro José de Moraes Martins, Virginia Genelhu, Marcia Mattos Gonçalves Pimentel, Bruno Miguel Jorge Celoria, Rogerio Fabris Mangia, Teresa Aveta, Cristoforo Silvestri, Vincenzo Di Marzo, Emilio Antonio Francischetti

**Affiliations:** 1 Laboratory of Clinical and Experimental Pathophysiology (CLINEX), Rio de Janeiro State University (UERJ), Rio de Janeiro, RJ, Brazil; 2 Pro-Rector for Research and Postgraduate Education, UNIGRANRIO, Duque de Caxias, RJ, Brazil; 3 Genetics Department, Rio de Janeiro State University (UERJ), Rio de Janeiro, RJ, Brazil; 4 Endocannabinoid Research Group, Institute of Biomolecular Chemistry, Consiglio Nazionale delle Ricerche, Pozzuoli NA, Italy; Laboratorio de Neurociencias Moleculares e Integrativas. Escuela de Medicina, División Ciencias de la Salud. Universidad Anáhuac Mayab. Mérida, Yucatán. México, MEXICO

## Abstract

The dysregulation of the endocannabinoid system is associated with cardiometabolic complications of obesity. Allelic variants in coding genes for this system components may contribute to differences in the susceptibility to obesity and related health hazards. These data have mostly been shown in Caucasian populations and in severely obese individuals. We investigated a multiethnic Brazilian population to study the relationships among the polymorphism 385C>A in an endocannabinoid degrading enzyme gene (*FAAH*), endocannabinoid levels and markers of cardiometabolic risk. Fasting plasma levels of endocannabinoids and congeners (anandamide, 2-arachidonoylglycerol, N-oleoylethanolamide and N-palmitoylethanolamide) were measured by liquid chromatography-mass spectrometry in 200 apparently healthy individuals of both genders with body mass indices from 22.5 ± 1.8 to 35.9 ± 5.5 kg/m^2^ (mean ± 1 SD) and ages between 18 and 60 years. All were evaluated for anthropometric parameters, blood pressure, metabolic variables, homeostatic model assessment of insulin resistance (HOMA-IR), adiponectin, leptin, C-reactive protein, and genotyping. The endocannabinoid levels increased as a function of obesity and insulin resistance. The homozygous genotype AA was associated with higher levels of anandamide and lower levels of adiponectin versus wild homozygous CC and heterozygotes combined. The levels of anandamide were independent and positively associated with the genotype AA position 385 of *FAAH*, C-reactive protein levels and body mass index. Our findings provide evidence for an endocannabinoid-related phenotype that may be identified by the combination of circulating anandamide levels with genotyping of the *FAAH* 385C>A; this phenotype is not exclusive to mono-ethnoracial populations nor to individuals with severe obesity.

## Introduction

The worldwide prevalence of overweight adults (body mass index [BMI] of 25 kg/m^2^ or greater) increased from 28.8 to 36.9% in men and from 29.8 to 38.0% in women between 1980 and 2013 [1]. Because of the established health risk and substantial increase in the prevalence of obesity, the death rate attributable to this increase has reached approximately three million individuals per year [2]. Subjects with a greater proportion of visceral adipose tissue have metabolically deleterious and life-threatening forms of obesity such as insulin resistance, type 2 diabetes mellitus, hypertension and dyslipidemia [3, 4]. The mechanism of obesity-induced major health issues is chronic inflammation in the white adipose tissue [5], and the development of white adipose tissue dysfunction. This may affect lipid handling and thus contribute to excessive fat accumulation in non-adipose tissues [6].

Endocannabinoids (eCBs) play an important role in these mechanisms. They are membrane phospholipid-derived mediators that act as endogenous ligands on the two known cannabinoid receptors (CB1 and CB2) that are present in the central nervous system and in the peripheral tissues and organs of many species [7]. Chronic CB1 receptor stimulation under different conditions induces glucose intolerance, stimulates metabolic inflammation and alters lipid metabolism in muscles, liver and adipose tissue [8]. The two best-studied eCBs are anandamide (AEA) and 2-arachidonoylglycerol (2-AG). Other related eCBs compounds, N-oleoylethanolamide (OEA) and N-palmitoylethanolamide (PEA), are generated from a common family of membrane phospholipids and share with AEA many of the enzymes for biosynthesis and degradation [9]. The eCBs are primarily inactivated by cellular reuptake and intracellular hydrolysis by the enzyme fatty acid amide hydrolase (FAAH)—mostly for AEA—and monoacylglycerol lipase, that is selective for 2-AG [10].

Increased levels of eCBs have been reported in obese men and women of different ages as well as in individuals with insulin resistance and type 2 diabetes [11–13]. While the literature has shown association between plasma AEA and 2-AG concentrations and BMI and intra-abdominal adiposity, there are also conflicting results. For example, increased plasma AEA and 2-AG levels have been reported in obese versus lean menopausal women [14], and elevated fasting levels of 2-AG but not AEA have been observed in men with increased visceral obesity [13]. Furthermore, the eCBs are differently associated with race and degrees of obesity [15].

There is also evidence that genetic variants of the endocannabinoid system (ECS) components could contribute to differences in the susceptibility to several human disorders including obesity and related metabolic disorders [16–19]. Due to the strong negative correlation between FAAH expression in adipose tissue and plasma eCB levels [12], one of the most frequently analyzed genetic variants has been the 385C>A polymorphism in *FAAH*. However, these studies have been conducted mostly in Caucasian populations [18, 19].

The present study investigates subjects with a wide BMI range via quantitative measurement of circulating eCBs and their related compounds as well as genotyping for the 385C>A polymorphism in *FAAH*. These variables are used to identify the obese phenotype associated with insulin resistance, dysregulation of adipocytokines (leptin and high molecular weight adiponectin) and cardiometabolic biomarkers. Furthermore, these data provide new and useful insights into the knowledge of eCBs. In contrast to previous studies, we studied a Brazilian multiethnic population characterized by intense interbreeding, but this has not been restricted to individuals with severe obesity.

## Materials and Methods

### Study sample

Our sample consisted of 200 subjects (100 normal weight and 100 obese individuals) who were recruited from the employees, students, residents and staff from a University Hospital (Pedro Ernesto University Hospital–Rio de Janeiro State University.) The Research Ethics Committee of Pedro Ernesto University Hospital approved this study without restrictions and subjects voluntarily agreed to participate and provided written informed consent. They were sequentially included according to the inclusion and exclusion criteria. Because we intended to study several quantitative variables and a polymorphism, we used this classical formula for sample size: n = z^2^/4δ, where n represents the number of subjects per group, Z is 1.96 and defines the 95% confidence interval (α = 0.05), and δ is the maximum tolerated deviation. Thus, as n = (1.96)^2^ / 4(0.1)^2^ = 96.04 per group.

Our inclusion criteria were: a) age between 18 and 60 years; b) an eutrophic group with a BMI ≥ 18.5 kg/m^2^ and < 25 kg/m^2^; and c) an obese group with BMI ≥ 30 kg/m^2^. Our exclusion criteria were: a) the presence of diabetes mellitus or current treatment with hypoglycemic drugs; b) the presence of stage 2 high blood pressure as defined by JNC VII or current treatment with antihypertensive drugs; c) previous cardiovascular condition (e.g., acute coronary syndrome, cerebrovascular event, or symptomatic peripheral arterial disease); d) known chronic conditions (e.g., chronic obstructive pulmonary, inflammatory bowel, liver, renal, hematological, psychiatric or autoimmune diseases, endocrinopathies, or malignant neoplasms); e) pregnancy or lactation; f) herbal or illegal drug use; g) use of drugs for weight loss in the previous 3 months; h) use of corticosteroids or nonsteroidal anti-inflammatory drugs or i) use of any drugs involved in carbohydrate or lipid metabolism.

### Anthropometric variables determination

The BMI was determined by dividing the weight in kilograms (kg) by the height in meters squared (m^2^). Weight was measured using an anthropometric scale (Filizola™—Brazil) with a precision of 0.1 kg in fasted subjects who wore light clothes and no shoes. Height was measured using a stadiometer with a precision of 0.5 cm. Waist and hip circumferences were measured with an inelastic tape while the subjects were standing up with the abdomen relaxed and the arms along the sides of the body. The waist circumference (WC) was measured in the middle of the distance between the iliac crest and the last rib. The hip circumference (HC) was measured in the higher posterior circumference of the buttocks. The waist-to-hip ratio (WHR) was obtained by dividing the WC by the HC.

### Blood pressure determination

Blood pressure was determined via an oscillatory method using an automatic blood pressure monitor (OMRON, model HEM-705CPINT). The cuff was selected according to the arm circumference. After resting for 5 minutes, the average of three measurements within 3 minutes was used. The mean blood pressure (MBP) was calculated as the diastolic blood pressure (DBP) plus one-third of the pulse pressure.

### Laboratory analyses

Venous blood samples were collected after a 12-hour night fasting period, and the aliquots were stored at -20 or -80 degrees Celsius. Fasting glucose was determined by the enzymatic hexokinase method.

Fasting insulin was measured with chemiluminescence. In this assay, one monoclonal antibody was coated on the surface of the microtiter wells and another monoclonal antibody, labeled with horseradish peroxidase, was used as the tracer. The insulin molecules in the sample were sandwiched between the two antibodies. Following the formation of the coated antibody-antigen-antibody-enzyme complex, the unbound antibody-enzyme labels were removed by washing. The horseradish peroxidase activity bound in the wells was assayed by chemiluminescence reactions. The related light unit of the reaction was proportional to the concentration of insulin in the sample. The intra- and inter-assay coefficients of variation were 1.5 and 4.9%, respectively.

The insulin resistant status was assessed by the homeostatic model assessment of insulin resistance (HOMA-IR) [20]. Insulin resistance was defined as a HOMA-IR ≥ 2.71, according to the threshold value obtained from a multiethnic population in the Brazilian Metabolic Syndrome Study [21]. The lipid profile (triglycerides and total and HDL-cholesterol) was obtained using enzymatic- colorimetric methods. The LDL-cholesterol was calculated using the Friedewald formula [22].

The levels of high sensitive C-reactive protein (hsCRP) were determined by high sensitive nephelometry in which particles consisting of a polystyrene core and a hydrophilic shell were used in order to link anti-hsCRP antibodies covalently. A dilute solution of test sample was mixed with latex particles coated with monoclonal anti-hsCRP antibodies with formation of an antigen-antibody complex with the latex particles. Light scattering, measured by a nephelometric procedure was proportional to the concentration of hsCRP present in the sample, calculated by using a calibration curve.

High molecular weight adiponectin (HMW adiponectin) was measured with ELISA (Millipore Biomanufacturing and Life Science Research–USA). This assay used concurrent capture of adiponectin molecules from samples to the wells of a microtiter plate coated with a monoclonal anti-human adiponectin antibodies, and binding of a second biotinylated monoclonal anti-human antibody to the captured molecules, washing of unbound materials from samples, binding of streptavidin-horseradish peroxidase conjugate to the immobilized biotinylated antibodies, washing of excess of free enzyme conjugates, and quantification of immobilized antibody-enzyme conjugates by monitoring horseradish peroxidase activities in the presence of the substrate 3,3’,5,5’-tetramethylbenzidine. The enzyme activity was measured spectrophotometrically by the increased absorbance at 450–590 nm after acidification of formed products. Human adiponectin in the sample was derived by interpolation from a reference curve generated in the same assay with reference standards of known concentrations of human adiponectin. The intra- and inter-assay coefficients of variation were 8.8 and 6.1%, respectively.

Leptin was measured with the Milliplex method (Luminex™—Human Metabolic Panel, Millipore Corp. USA), which used color-code microspheres with two fluorescent dyes. Distinctly colored bead sets of polystyrene microspheres were coated with a specific capture antibody. After leptin from a test sample was captured by the bead, a biotinylated detection antibody was introduced. The reaction mixture was then incubated with streptavidin-peroxidase conjugate, the reporter molecule, to complete the reaction on the surface of each microsphere. Each individual microsphere was identified and the result of its bioassay was quantified based on fluorescent reporter signals. The intra- and inter-assay coefficients of variation were 9 and 8%, respectively.

### Endocannabinoids and congeners determination

Whole blood samples were collected in evacuated glass tubes that contained EDTA. The samples were centrifuged to separate plasma from blood cells, and the plasma was decanted and stored in 1 mL aliquots at -80°C prior to plasma lipid extraction. For each sample, 0.5 mL plasma was added to a polypropylene plastic tubes that contained 2.0 mL chloroform (CHCl_3_) and 1.0 mL methanol (MeOH). To this mixture, aliquots of 10pmol d5-2-arachidonoylglycerol and 5pmol d8-arachidonoyl-ethanolamide were added. The vial contents were vortex mixed for 30 seconds and centrifuged at 10°C (1400 x g for 10 minutes). The organic layer was carefully removed while avoiding the aqueous layer and dried under a stream of nitrogen (N_2_) gas. The aqueous phase was then extracted two more times with an equal volume of CHCl_3_, and the organic phase was subsequently dried again and pooled with the first dried organic phase. The lipid extract was then re-solubilized in 100 microliters of 2:1 CHCl_3_:CH_3_OH and pre-purified on silica columns. The eluate from the column with 9:1 CHCl_3_:CH_3_OH was dried and analyzed. Quantitative analysis for AEA and 2-AG as well as other congeners was performed on a Shimadzu liquid chromatographer coupled with an atmospheric pressure-chemical ionization-single quadrupole mass spectrometer using positive ion analysis mode. The values for eCBs and congeners were subsequently calculated using ratios of the deuterated internal standards to calculate the absolute concentrations.

### Molecular analysis

Genomic DNA was isolated from the peripheral blood leukocytes, and genotyping of the *FAAH* 385C>A variant was performed with bidirectional sequencing using the Big Dye Terminator v3.1 Kit (Life Technologies Inc., Foster City, CA). The sequencing of *FAAH* was conducted on an ABI 3130 genetic analyzer automatic sequencer (Life Technologies Inc.), and the sequences were evaluated using Chromas Lite 2.1.1 software (Technelysium Pty Ltd., South Brisbane, Australia).

### Statistical analysis

Kolmogorov-Smirnov and Levene tests were used to test the normality of the distribution and homogeneity of variances, respectively. Student’s t and Mann-Whitney tests were used for variables with and without a normal distribution, respectively. Weighed contrast ANOVA and Jonckheere-Terpstra tests were used to identify the mean and median trends, respectively. The partial correlation coefficient was used to analyze correlations among variables with a previous log transformation of the variables without normal distribution and Bonferroni adjustment for multiple testing. The Hardy-Weinberg equilibrium of the genotype frequencies was tested using the chi-squared test. A logistic regression analysis was used to determine the odds ratios of the association between the genotypes and obese and insulin-resistant phenotypes. A stepwise multiple linear regression model was used to analyze clinical correlations of endocannabinoids with other variables.

In all statistical analyses, a two-tailed P value < 0.05 was considered significant. All analyses were performed using statistical software PASW Statistics v. 18 (IBM SPSS, Inc.).

## Results

The data distributions in the total sample as well as eutrophic and obese subsets are shown in Table 1.

**Table 1:** Characteristics of the study population.

Variables	Total (n = 200)	Eutrophics (n = 100)	Obeses (n = 100)	*P* value
	M (n = 100)/F (n = 100)	M (n = 50)/F (n = 50)	M (n = 50)/F (n = 50)	
**Age (years)**	35.0 ± 10.3	32.4 ± 9.8	37.6 ± 10.2	< 0.001
**Body mass index (kg/m** ^**2**^ **)**	29.1 ± 7.8	22.5 ± 1.8	35.7 ± 5.5	< 0.001
**Waist circumference (cm)**	94.7 ± 19.3	79.0 ± 7.2	110.4 ± 14.0	< 0.001
**Waist-to-hip ratio**	0.89 ± 0.10	0.84 ± 0.10	0.94 ± 0.08	< 0.001
**Systolic blood pressure (mmHg)**	123.0 ± 14.2	117.4 ± 11.4	128.6 ± 14.6	< 0.001
**Diastolic blood pressure (mmHg)**	75.8 ± 9.7	71.7 ± 7.8	79.9 ± 9.8	< 0.001
**Mean blood pressure (mmHg)**	91.5 ± 10.7	86.9 ± 8.3	96.0 ± 11.0	< 0.001
**Fasting glucose (mmol/L)**	5.02 ± 0.52	4.88 ± 0.43	5.15 ± 0.56	< 0.001
**Total cholesterol (mmol/L)**	4.82 ± 1.05	4.71 ± 0.96	4.93 ± 1.13	0.13
**HDL-cholesterol (mmol/L)**	1.36 ± 0.37	1.50 ± 0.35	1.21 ± 0.33	< 0.001
**LDL-cholesterol (mmol/L)**	2.95 ± 0.94	2.79 ± 0.82	3.11 ± 1.01	0.02
**Triglycerides (mmol/L)**	0.96 (0.71; 1.38)	0.82 (0.67; 1.01)	1.20 (0.87; 1.68)	< 0.001
**Fasting insulin (pmol/L)**	54.17 (36.11; 90.28)	38.19 (23.61; 52.08)	87.50 (58.33; 111.12)	< 0.001
**HOMA-IR**	1.74 (1.07; 3.00)	1.17 (0.77; 1.64)	2.78 (1.87; 3.60)	< 0.001
**Leptin (ng/mL)**	9.17 (2.79; 19.83	3.82 (1.35; 9.16)	17.34 (9.07; 30.60)	< 0.001
**HMW adiponectin (μg/mL)**	3.04 (1.61; 4.88)	4.11 (2.44; 5.65)	2.20 (1.20; 3.54)	< 0.001
**hsCRP (nmol/L)**	18.09 (8.57; 47.62)	10.47 (7.61; 19.04)	34.28 (16.19; 68.57)	< 0.001
**Anandamide (pmol/mL)**	2.50 (1.91; 3.32)	2.07 (1.83; 2.94)	2.82 (2.13; 3.81)	< 0.001
**2-arachidonoylglycerol (pmol/mL)**	5.72 (3.60; 8.87)	5.86 (3.67; 8.54)	5.59 (3.43; 9.18)	0.66
**N-palmitoylethanolamide (pmol/mL)**	220.97 (49.68; 283.89)	233.12 (44.98; 283.82)	212.73 (53.51; 286.32)	0.27
**N-oleoylethanolamide (pmol/mL)**	20.16 (15.52; 25.49)	17.95 (14.23; 22.76)	22.65 (16.67; 28.57)	< 0.001

Values are mean ± SD for variables with normal distribution or median (25^th^; 75^th^ percentiles) for variables without normal distribution.

*P* value for differences between variables in eutrophic and obese subjects.

M, male gender; F, female gender; HOMA-IR, homeostasis model assessment of insulin resistance; HMW adiponectin, high molecular weight adiponectin; hsCRP, high sensitive C reactive protein.

**Fig 1:**
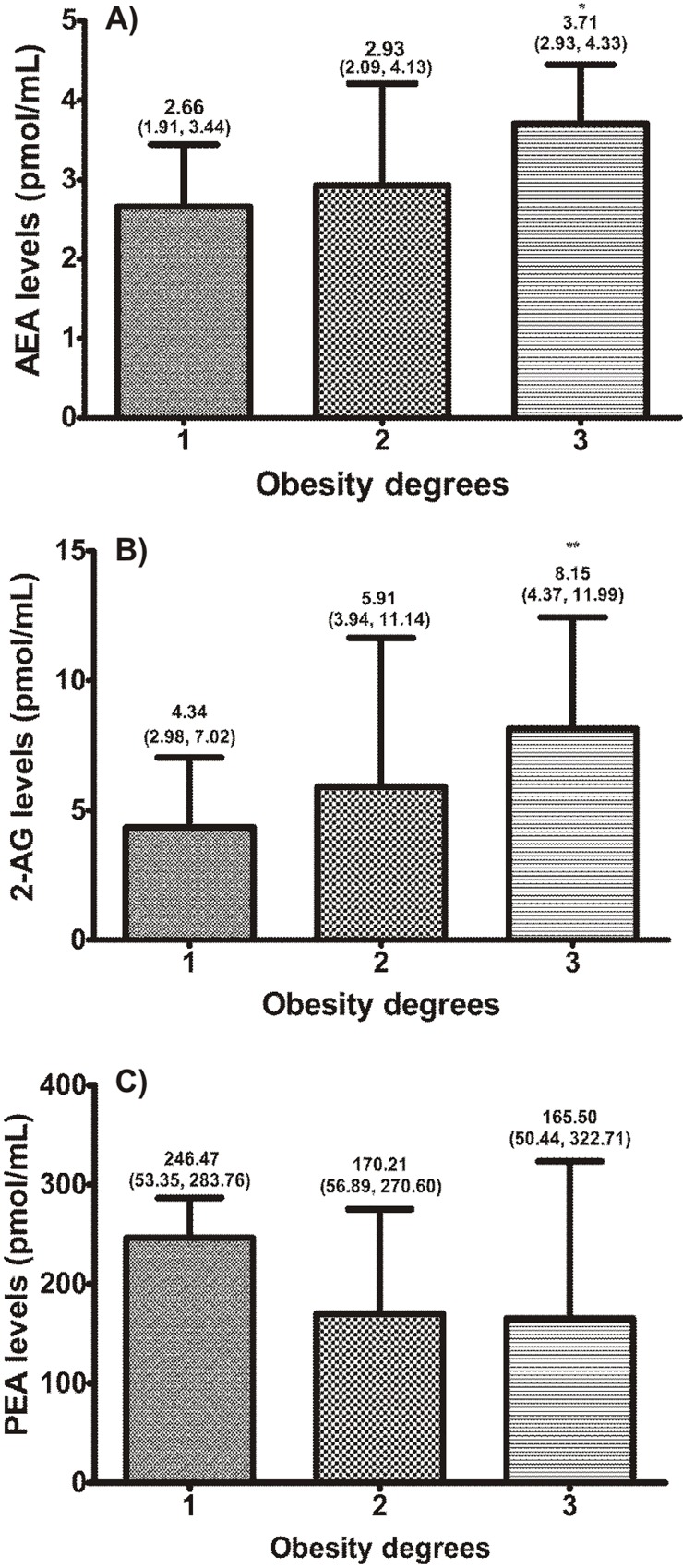
Relationships of obesity degrees with (A) AEA, (B) 2-AG and (C) PEA levels. Values are medians (25^th^, 75^th^ percentiles). Figures show medians and interquartile range. *P* values for trends of medians (Jonckheere-Terpstra test). *P = 0.04; **P = 0.003. AEA, anandamide; 2-AG, 2-arachidonoylglycerol; PEA, N-palmitoylethanolamide.

The variable distribution along the three degrees of obesity is shown in Fig 1 (there are 54 subjects in the first degree, 32 subjects in the second degree and 14 subjects in the third degree).

There was a significant increasing trend in AEA (Fig 1A), 2-AG (Fig 1B), WC (*P*<0.001), insulin (*P* = 0.005), HOMA-IR (*P* = 0.004), leptin (*P* = 0.008), hsCRP (*P* = 0.002), along the three degrees of obesity. PEA had no significant trend (Fig 1C).

**Fig 2:**
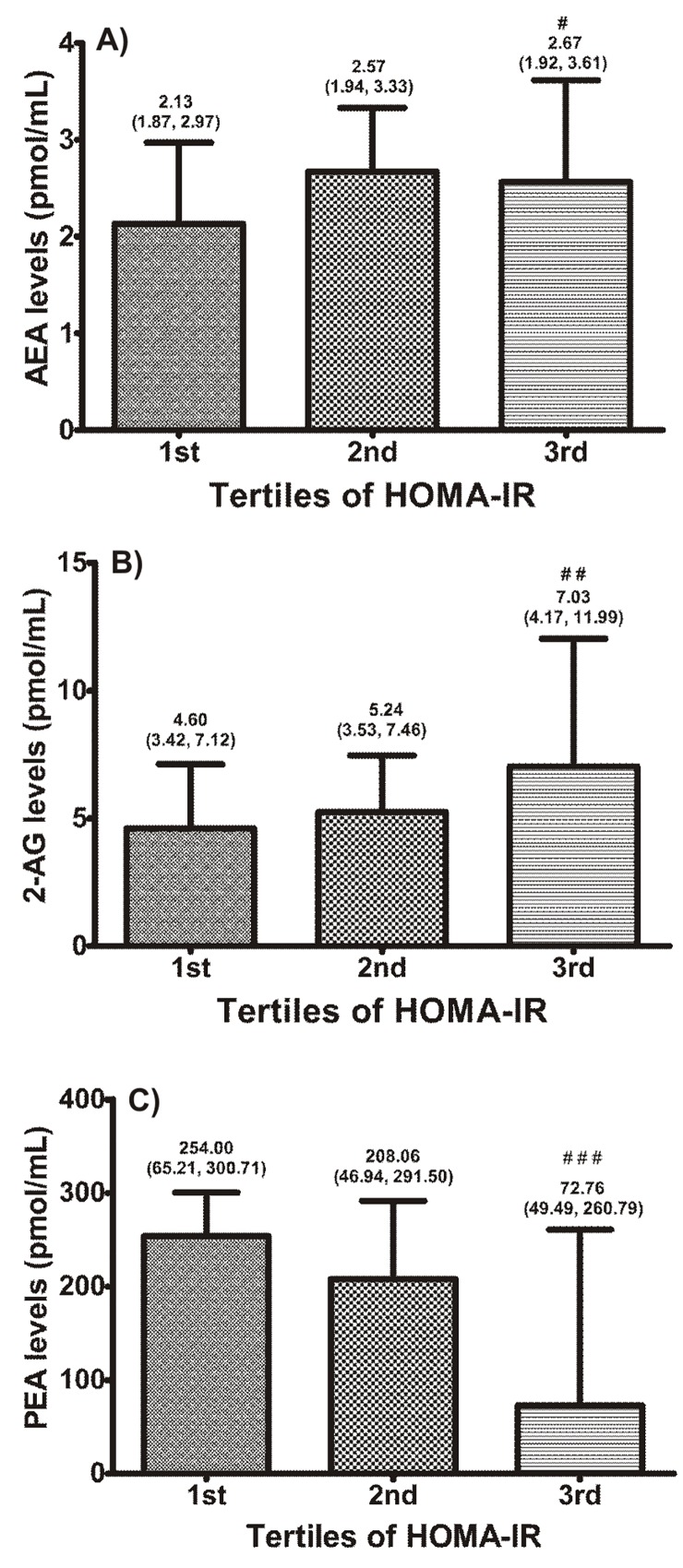
Relationships of HOMA-IR tertiles with (A) AEA, (B) 2-AG and (C) PEA levels. Values are medians (25^th^, 75^th^ percentiles). Figures show medians and interquartile range. *P* values for trends of medians (Jonckheere-Terpstra test). ^#^ *P* = 0.03; ^##^ *P* = 0.006; ^###^ *P* = 0.004. AEA, anandamide; 2-AG, 2-arachidonoylglycerol; PEA, N-palmitoylethanolamide.

Tertiles of HOMA-IR were created to represent the progressive increments of insulin resistance in the eutrophic and obese subjects as a group (Fig 2). Along the increasing insulin resistance tertiles, the variables AEA (Fig 2A), 2- AG (Fig 2B), BMI, WC, WHR, SBP, DBP, MBP, glucose, triglycerides, insulin, leptin, hsCRP (all *P*<0.001) and OEA (*P* = 0.03), exhibited a significant increasing trend, whereas HDL-cholesterol (*P*<0.001), HMW adiponectin (*P*<0.001) and PEA (Fig 2C) decreased. Age, total cholesterol and LDL-cholesterol had no significant trend.

Age- and sex-adjusted partial correlation coefficients were determined to demonstrate the main correlations of circulating endocannabinoids (Table 2).

**Table 2:** Main correlations of circulating endocannabinoids.

Variable	AEA	PEA
**Body mass index**	*r* = 0.34	NS
**Wait circumference**	*r* = 0.31	NS
**HOMA-IR**	NS	*r* = -0.25
**Leptin**	NS	*r* = -0.28
**High sensitive C reactive protein**	*r* = 0.33	NS

Values are age- and sex-adjusted partial correlations with Bonferroni adjustment for multiple testing for a significance level < 0.05.

AEA, anandamide; PEA, palmitoylethanolamide; NS, non-significant.

### Genotype frequency and association with obese and insulin resistant phenotypes

The genotype frequencies for polymorphism 385C>A in *FAAH* were: CC, 114 subjects (63 eutrophics and 51 obeses); CA, 72 subjects (32 eutrophics and 40 obeses); and AA, 14 subjects (5 eutrophics and 9 obeses). The genotype distribution followed the Hardy-Weinberg equilibrium. There was no significant association with obese or insulin resistant phenotype.

### Association of polymorphisms with eCBs, adipocytokine levels and cardiometabolic variables

The homozygous genotype AA for the polymorphism 385C>A in *FAAH* was associated with higher levels of AEA versus the wild homozygous CC and heterozygotes combined. In the obese group, the homozygous genotype AA was also associated with higher levels of OEA and lower levels of HMW adiponectin (Table 3).

**Table 3:** Association of polymorphism *FAAH* 385C>A with AEA, OEA and HMW adiponectin levels.

		Whole group			Eutrophics			Obeses	
Variables	AA	CC+CA	*P*	AA	CC+CA	*P*	AA	CC+CA	*P*
	(n = 14)	(n = 186)	value	(n = 5)	(n = 95)	value	(n = 9)	(n = 91)	value
**AEA**	3.36	2.45	0.02	2.76	2.07	0.04	4.13	2.78	0.02
**(pmol/mL)**	(2.10, 5.05)	(1.90, 3.28)		(2.02, 2.93)	(1.73, 2.94)		(2.54, 4.97)	(2.05, 3.63)	
**OEA**	25.67	20.09	0.12	17.34	18.21	0.68	28.51	22.21	0.02
**(pmol/mL)**	(16.81, 35,13)	(15.48, 25.08)		(13.99, 17.84)	(14.30, 22.90)		(22.93, 38.16)	(16.33, 27.99)	
**HMW adip**	1.86	3.09	0.10	4.09	4.12	0.83	1.36	2.33	0.02
**(μg/mL)**	(1.24, 3.93)	(1.67, 4.92)		(3.88, 4.42)	(2.43, 5.72)		(1.13, 1.92)	(1.21, 3.59)	

Values are medians (percentiles 25^th^, 75^th^).

*P* values for differences in variables levels between AA and CC + CA genotypes (Mann-Whitney test).

Variables are age- and sex-adjusted.

AEA, anandamide; OEA, N-oleoylethanolamide; HMW adip, high molecular weight adiponectin.

The other variables had no significant relationships with the polymorphism. In a model of multiple linear regression analysis for AEA, the independent positive associations with AEA were the presence of the homozygous genotype AA in position 385 of *FAAH*, hsCRP levels and BMI (Table 4).

**Table 4:** Stepwise multiple linear regression of anandamide in the entire sample.

Variables	b	EP	β	*P* value	95% CI
**Constant**	0.69	0.17	-	< 0.001	0.35 a 1.03
**Gender**	0.06	0.06	0.07	0.30	-0.05 a 0.17
**Age (years)**	0.001	0.003	-0.002	0.98	-0.01 a 0.01
**BMI (kg/m** ^**2**^ **)**	0.01	0.004	0.21	0.007	0.003 a 0.02
**hsCRP (nmol/L)**	0.10	0.03	0.24	0.002	0.04 a 0.16
**AA genotype**	0.39	0.11	0.24	< 0.001	0.19 a 0.60

b, regression coefficient; EP, standard error of regression coefficient b; β, standard regression coefficient. *P* value, statistical significance of regression coefficient b; 95% CI, 95% confidence interval for regression coefficient b.

BMI, body mass index; hsCRP, high sensitive C reactive protein; AA genotype, presence of homozygous genotype AA in position 385 of *FAAH*.

## Discussion

Predicting obesity-associated health hazards is of paramount importance. This can be done by characterizing phenotypically and genetically at-risk individuals who would benefit from personalized monitoring and care. In the present study, we reported that AEA was independent and positively associated with the homozygous genotype AA of the *FAAH* 385 variant, hs-CRP and BMI.

The activation of the ECS in obesity is associated with increased concentrations of endogenous cannabinoids in various tissues, plasma and saliva [7, 14, 23]. Here, similar to what has already been reported [12,14], we show that circulating levels in the blood of AEA and OEA, but not 2-AG, were significantly increased in obese subjects versus eutrophic controls (Table 1). Furthermore, plasma levels of AEA, 2-AG and OEA were positively correlated with WC, WHR, HOMA-IR, hsCRP and leptin (Table 2). There was a progressive and significant increase in AEA and 2-AG along the three degrees of obesity (Fig 1A and 1B).

Of potential interest to our findings, increased plasma levels of AEA and 2-AG were recently shown to be inversely associated with coronary endothelial function in obese subjects [24]. This observation is suggestive of adverse effects of these bioactive molecules on the coronary endothelium that may anticipate unfavorable outcomes in conditions such as mild to severe forms of coronary artery disease.

Still, regarding the AEA and 2-AG levels, our results are in apparent contrast with those published in the classic paper by Côté et al. [13]. They studied 62 Caucasian males and showed that circulating levels of 2-AG, but not AEA, correlated positively with BMI, waist girth, intra-abdominal adiposity and insulin levels. In addition, AEA plasma levels were negatively correlated with intra-abdominal adiposity. Such differences might be due to ethnoracial and gender characteristics as well as the degree of obesity.

In this current study, we included individuals who exhibited higher BMI and waist circumference values than those of Côte et al (35.4 ± 7.1 versus 27.4 ± 4.5 kg/m²; 110.4 ± 14.0 versus 94.6 ± 12.3 cm). Moreover, our population was multiethnic and both genders were equitably distributed (100/100). Recently, Mallipedhi et al. showed that obese subjects of both genders submitted to bariatric surgery exhibited preoperatively higher levels of AEA, OEA, and PEA, but not 2-AG in the female group versus men. Additionally, reductions of AEA and PEA levels were reported only in females who lost weight postoperatively [25]. The reasons for these differences are still elusive and could be related to several factors such as different bioactivities in insulin resistance between genders and possible interactions between sex hormones and eCBs [26, 27]. Therefore, further studies are necessary to understand the impact of gender and ethnic dissimilarities in relation to eCBs.

Additional analysis of our data revealed an increasing trend of AEA and 2-AG levels along the HOMA-IR tertiles in the entire sample (Fig 2A and 2B). Moreover, waist circumference also progressively increased with increases in HOMA-IR. This highlights the important relationship between visceral adiposity, eCBs and insulin resistance. This also corroborates the higher levels of AEA and 2-AG in visceral versus subcutaneous adipose tissue in mice with diet-induced obesity [28] and altered levels of 2-AG in the adipose tissue of obese subjects following weight loss [29].

Recently, Abdulnour et al [30] evaluated the differences in circulating levels of AEA and 2-AG in a group of obese postmenopausal women before and after weight loss. In that study, higher circulating levels of 2-AG, but not AEA, were found in insulin-resistant but not in insulin-sensitive obese women. Altogether, these results are validated by experimental data showing that insulin-induced regulatory patterns of the ECS are lost in insulin-resistant adipocytes. This results in an inability to reduce intracellular eCBs. This peripheral dysregulation of ECS upregulates the expression of CB1 receptor activity and reduces glucose uptake in skeletal muscle [28, 31].

We also found similarities between the associations of OEA and AEA levels with cardiometabolic risk variables (Table 2). This finding can be justified by the strong direct association between the two compounds because of their common biosynthesis and degradation pathways despite actions on different receptors. While AEA acts on CB1 and CB2, OEA primarily activates peroxisome proliferator-activated receptor-α (PPAR-α)—a nuclear receptor involved in carbohydrate and lipid metabolism [32]. The activation of PPAR-α by OEA, unlike the effects of CB1 receptor activation, inhibits lipolysis in liver and adipose tissue, reduces food intake, and induces satiety [33]. In addition, intestinal levels of AEA and OEA appear to be inversely correlated with food intake. It may be possible that AEA and OEA could act in a coordinated manner in the gastrointestinal tract [34] and that the activation of PPAR-α by OEA would not be sufficient to overcome the effects of CB1 receptor activation by AEA [7].

Contrariwise, we found that PEA levels decreased along the three degrees of obesity as well as along increasing tertiles of HOMA-IR (Fig 2C). PEA does not act on surface cannabinoid receptors (CB1 and CB2); it binds to and enhances transcription activity of PPARα [35]. Fibrates are drugs that also activate PPAR-α receptors and are used to control dyslipidemias and improve metabolic profile. Thus, we can assume that the decreasing levels of PEA with progressive increases in adiposity and, more importantly, insulin resistance, would expose subjects with this profile to higher cardiometabolic risk. This is further corroborated by the findings of Abdulnour et al. who showed increasing levels of PEA following weight loss in obese postmenopausal women [30]. The demonstration of inverse correlation of PEA with HOMA-IR and hsCRP strengthens the hypothesis of its beneficial role in the metabolic profile.

### Relationships between the *FAAH* 385C>A polymorphism, cardiometabolic variables, eCBs and congeners

In 2004, Chiang et al. demonstrated that subjects with *FAAH* 385 A/A missense polymorphism have approximately half the FAAH enzymatic activity and protein expression than wild-type subjects [36]. This same group is now performing a population-based study of 1,688 subjects of distinct racial backgrounds. They have shown that the homozygous *FAAH* 385 A/A genotype was significantly associated with overweight and obesity in Caucasian subjects of European ancestry and in Negroids, but not in Asians. Additionally, the median BMI for all subjects of that study was significantly greater in the *FAAH* 385 A/A genotype group than the heterozygote and wild-type groups [37].

Subsequently, Monteleone et al. [38] also associated a cDNA 385 C to A missense polymorphism in the *FAAH* of Caucasian women with overweight/obesity, but not with binge eating disorder. We failed to replicate these findings. However, and concurring with our findings, no association was found between the *FAAH* A allele and BMI, WC, WHR, HOMA-IR, and other risk factors in a relatively large population-based sample of 5,801 Danish Caucasians [39]. Possible explanations for these discordances might be the size of the study samples and the ethnic characteristics of individuals. Of note, these characteristics varied from well-defined racial groups of Caucasians, Negroids and Asians to a heterogeneous interethnic Brazilian population who progressively became more homogeneous by constant interbreeding. In addition, there may be other mechanisms that interfere with the expression and function of the variant protein such as the impact of various gene-gene/gene-environment interactions of the study subjects.

A striking association was identified between increased levels of AEA and the AA homozygous genotype in our study sample covering a wide range of adiposity values (Table 3). This finding has also been accompanied by significantly higher levels of OEA and lower concentrations of adiponectin in obese individuals with the AA genotype. These results also suggest a functional FAAH enzyme mutation in subjects with polymorphism 385C>A in *FAAH* [40], and replicate experimental animal models where pharmacological inhibition or genetic deletion of *FAAH* enhances the levels of AEA [41, 42]. On the other hand, the inverse association between AEA levels and adiponectin may reflect the CB1 receptor overstimulation by AEA with subsequent reduction of adiponectin expression in adipocytes as previously demonstrated by Matias et al [31]. Conversely, the treatment of mouse adipocytes with rimonabant, an antagonist of CB1 receptor, was associated with significantly increased levels of adiponectin mRNA versus control cells [43].

Partially contrasting our findings, Sipe et al. [44] showed in 96 severely obese subjects with BMI of ≥40 kg/m^2^ and 48 normal weight subjects with BMI of ≤26 kg/m^2^ significantly increased levels of AEA and related compounds in carriers of the *FAAH* 385 A mutant alleles than wild-type *FAAH* controls. In that study, however, this difference in AEA levels was only evident when severely obese subjects carrying the 385A allele were compared to wild-type eutrophic controls (CC homozygotes). Thus, our finding of high AEA levels in homozygous carriers of the A allele in obese individuals with varying degrees of adiposity is the first evidence that documents the role of this genetic variant as an independent predictor of ECS activation in a population with such characteristics (Table 4). Also relevant was the independent positive association between hsCRP levels and BMI as a function of AEA concentrations.

### Strengths and limitations

An important and original aspect of our study is the multiethnic characteristic of the cohort. Another important aspect is the expansion of knowledge regarding the influence of genetic variants on biomarkers of the ECS. Although biochemical evidence of the dysfunction of this enzyme has previously been reported, there are relatively few clinical studies describing this association. Additionally, the subjects included in this current study were apparently healthy individuals who have not been using any drugs involved in carbohydrate or lipid metabolism control or hypertension treatment.

The quantification and evaluation of the distribution of fat mass and insulin resistance were performed by indirect measurements of these parameters. To circumvent these biases, we chose simple and well-established surrogate outcomes, which have been widely used in population studies. Finally, our study was cross-sectional; therefore, it was not designed to infer causality, but rather associations. Thus, prospective studies are needed to clarify these questions.

## Conclusions

Our findings suggest that plasma levels of AEA in combination with genotyping of the *FAAH* 385 variant and hsCRP may identify an endocannabinoid-related obesity phenotype in a population with complex interethnic admixture. This is not exclusive to severely obese individuals, and it might make them more susceptible to major life-threatening forms of obesity.
